# Geographic Influence on Secondary Metabolite Profiles in Leaves of the Endemic *Agathosma betulina* (P.J. Bergius) Pillans. in the Western Cape Province, South Africa

**DOI:** 10.3390/ijms27104486

**Published:** 2026-05-16

**Authors:** Nompumelelo H. Mnisi, Rotondwa P. Gunununu, Luvolwethu Dukashe, Manaka J. Makgato, Azwimbavhi R. Mulidzi, Callistus Bvenura, Ngwatshipane M. Mashabela

**Affiliations:** 1 Department of Crop Sciences, Tshwane University of Technology, Private Bag X680, Pretoria 0001, South Africa; mnisihappiness8@gmail.com (N.H.M.); gunununurp@tut.ac.za (R.P.G.); dukashel@arc.agric.za (L.D.); 2 Agricultural Research Council Infruitec-Nietvoorbij, Private Bag X5026, Stellenbosch 7599, South Africa; makgatom@arc.agric.za (M.J.M.); mulidzir@arc.agric.za (A.R.M.); 3 Department of Horticulture, Faculty of Applied Sciences, Cape Peninsula University of Technology, Bellville 7535, South Africa; bvenurac@cput.ac.za

**Keywords:** *Agathosma betulina*, medicinal plant, flavonoids, phenolic acids, secondary metabolites

## Abstract

The role of medicinal plants in primary healthcare and livelihoods around the world is both ancient and well-documented. *Agathosma betulina* (P.J. Bergius) Pillans, commonly known as ‘buchu’, has long been utilised in traditional medicine as a household remedy for various ailments and is also valued for its essential oils in the cosmetics and pharmaceutical industries. This study aimed to profile and quantify the secondary metabolites in buchu using ultra-performance liquid chromatography quadrupole time-of-flight combined with mass spectrometry (UPLC-QTOF-MS) techniques, whereby plant material from three distinct locations in the Western Cape Province, Groot Winterhoek, Citrusdal, and Cederberg, was collected. A total of 32 maker compounds were identified from buchu leaves. The results revealed a significant location-dependent variation in the accumulation of multiple classes of phytochemicals, including phenolic acids, flavonoids, saponins, terpenoids, oligosaccharides, vitamins, and steroids. Citrusdal samples had the most bioactive compounds compared to the Cederberg and Groot Winterhoek. Citrusdal had the highest flavonoid levels, while Cederberg samples were the richest in phenolic acids and Groot Winterhoek was dominant in iridoid glycoside levels. Principal component analysis (PCA) revealed distinct clusters corresponding to the three different regions, confirming chemical differences. Elucidating the distribution of secondary metabolites in this species may provide new information for possible medicinal and pharmacological uses, such as the creation of novel and enhanced organic medications and food products. These results will aid in selecting a buchu chemotype with optimal attributes for the intended therapeutic application, helping to protect wild populations from over-exploitation through cultivation.

## 1. Introduction

Medicinal plants have a long and well-established history of use in primary healthcare and livelihoods worldwide. South Africa is among the world’s most biodiverse nations, with wild resources significantly contributing to the country’s economy and to the support of livelihoods in many communities of indigenous people, either as a source of income or direct use [1]. Buchu, botanically known as *Agathosma betulina* (P.J. Bergius) Pillans, is a fragrant flowering plant native to the lower elevation mountain fynbos habitats of the Western Cape Province, South Africa [2]. The plant is a perennial shrub belonging to the *Rutaceae* family. Buchu is a significant aspect of indigenous Khoi-San communities, used for centuries as a general medication, a tonic and for ceremonial purposes in the Cape [3]. It is among the few Southern African medicinal plants that have gained international market presence due to colonial interests. The commercial and therapeutic significance of *A. betulina* stems largely from its secondary metabolites, which are biologically active compounds such as monoterpenes, sesquiterpenes, terpenoids and flavonoids [4,5].

Buchu contains flavonoids (diosmin, hesperidin and rutin), mucilage, phenolics and other non-volatile marker compounds which have strong antioxidant qualities [6,7]. Its therapeutic properties perpetuate its use as a diuretic and as an anti-inflammatory and antimicrobial agent [7]. Several biological screenings have been performed to authenticate the use of buchu in ethnomedicine, with numerous pharmacological properties being reported [5,7]. Skosana et al. (2014) [8] reported that buchu extracts may help treat benign prostatic hyperplasia, prostate inflammation, and urinary tract infections when combining the anti-inflammatory and antioxidant properties. These compounds not only contribute to the plants’ distinctive aroma and pharmacological activity but also play essential ecological roles in plant defence and environmental adaptation [9]. Unlike primary metabolites, the synthesis and concentration of secondary metabolites are highly responsive to external factors, including altitude, temperature, soil composition, and precipitation [10,11]. Studies on other medicinal plants have demonstrated significant intraspecific variations in secondary metabolite contents across different geographic regions [12]. In the case of *A. betulina*, preliminary findings suggest that its chemical composition may also vary with geographic and environmental gradients within the Western Cape [13]. Such geographic variations hold important implications for standardisation in the medicinal plant trade, as well as for the conservation of genetic and phytochemical diversity in wild populations. Understanding how geographic and environmental variables influence secondary metabolite production in *A. betulina* is critical for both ecological and commercial reasons. From a conservation standpoint, the knowledge of phytochemical variation can inform the management of wild populations and promote the sustainable harvesting of chemically rich genotypes [14]. From a commercial perspective, identifying regions that yield plants with high concentrations of desirable compounds can enhance cultivation strategies and product consistency.

Therefore, this study aimed to investigate geographic variation in the secondary metabolite profiles of *A. betulina* across multiple populations in the Western Cape. This was achieved by analysing leaf samples from diverse habitats, through an untargeted metabolomics-based ultra-performance liquid chromatography quadrupole time-of-flight combined with mass spectrometry (UPLC-QTOF-MS). We sought to determine the extent of phytochemical differentiation and identify environmental factors that may drive this variation. The findings contribute to a broader understanding of the complex interplay between geography, environment, and plant chemistry, with potential applications in conservation, pharmacognosy, and the cultivation of high-value medicinal plants.

## 2. Results and Discussion

### 2.1. Soil Analysis

Soil samples from the three study sites showed notable variation in both physical and chemical properties (Table 1). Understanding these properties provides a valuable perspective on how soil characteristics influence the quality and phytochemical composition of medicinal plants, thereby supporting the interpretation of the metabolite profiles observed in this study.

The soil analysis showed that although all three sites were sandy, they differed notably in nutrient status, organic matter content, and acidity. Groot Winterhoek soils were highly acidic (pH 3.7) with low nutrient levels (NO_3_^−^ was <0.36 mg kg^−1^, P = 6.3 mg kg^−1^, and NH_4_^+^ = 2.3 mg kg^−1^) and limited water-holding capacity (29.2% at 10 kPa and 127.5 mm/m), indicating stressful conditions for plant growth. In contrast, Citrusdal soils were more fertile (pH 4.4) with higher organic carbon (1.69%), organic matter (2.91%), phosphorus (15.1 mg kg^−1^), and ammonium (8.5 mg kg^−1^), along with improved cation exchange capacity (2.99 cmol kg^−1^) and calcium saturation (47.83%), suggesting a more supportive environment. Cederberg soils were intermediate (pH 4.4); however, they exhibited relatively balanced nutrient composition and the highest cation exchange capacity (3.50 cmol kg^−1^). These variations indicate that soil fertility and acidity differences among sites will likely affect *A. betulina* secondary metabolite production.

### 2.2. UPLC-QTOF-MS Identification and Characterisation of the Bioactive Metabolites

**Figure 1:**
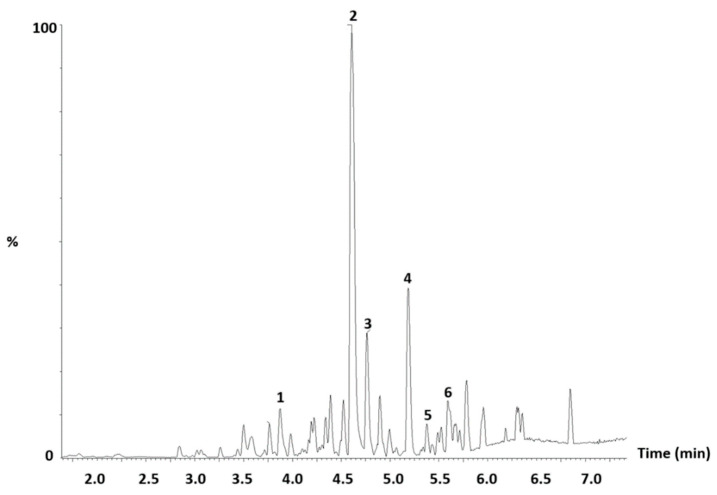
Base peak intensity (BPI) chromatogram of *Agathosma betulina*. [1] = Chlorogenic acid 353.0878 *m*/*z*, [2] = Rutin *m*/*z* 609.14636 *m*/*z*, [3] = Quercetin 3-galactoside 463.08875 *m*/*z*, [4] = Hesperidin 609.18262 *m*/*z*, [5] = Diosmin 607.16772 *m*/*z*, and [6] = epi-12-Palmatoside G 491.19247 *m*/*z*.

A representative base peak intensity (BPI) chromatogram obtained in negative ionisation mode is displayed in Figure 1. Table 2 shows the identification and average quantification of bioactive metabolites in *A. betulina* from the three locations using UPLC-QTOF-MS. A total of 32 compounds were tentatively identified from the leaves of buchu, with retention times ranging from 2.817 to 6.356 min. The compounds were identified according to their molecular formula, retention time and their fragment ions in comparison with data from the literature.

**Figure 2:**
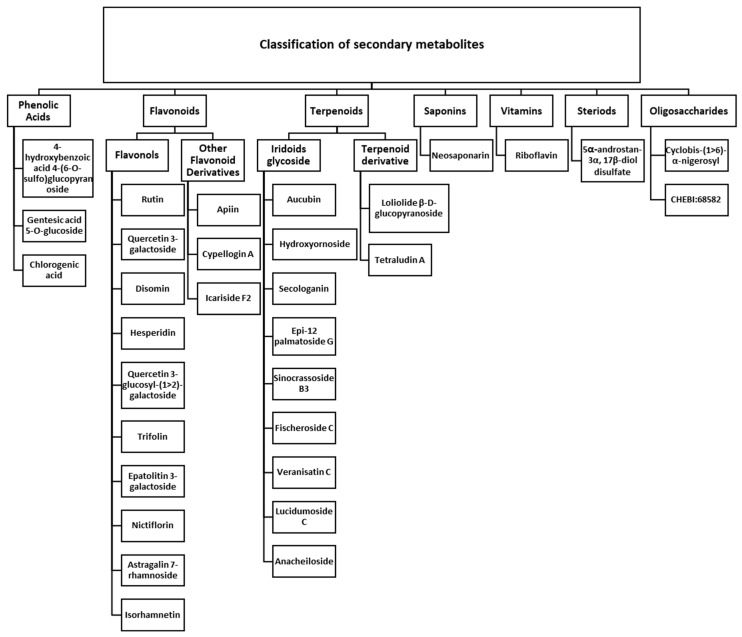
Structural classification of identified secondary metabolites in *Agathosma betulina* using UPLC-QTOF-MS analysis.

The phytochemical analysis of *A. betulina* in the current study demonstrated a rich and diverse composition of secondary metabolites, with flavonoids representing the predominant class (Figure 2). The identified secondary metabolites were categorised into seven classes: phenolic acids, flavonoids, terpenoids, saponins, vitamins, steroids, and oligosaccharides.

#### 2.2.1. Phenolic Acids

Three phenolic acids were identified: 4-hydroxybenzoic acid 4-(6-O-sulfo) glucopyranoside, gentesic acid 5-O-glucoside, and chlorogenic acid, as presented in Table 2 and Figure 2. Among these, 4-hydroxybenzoic acid 4-(6-O-sulfo) glucopyranoside exhibited the highest concentration in samples from Citrusdal, reaching 4.32 mg/g. In contrast, the highest levels of gentesic acid 5-O-glucoside (29.03 mg/g) and chlorogenic acid (14.65 mg/g) were detected in specimens that were collected from the Cederberg mountains. The high levels of phenolic acids found in samples from the Cederberg region are likely due to a combination of environmental stressors, such as high light levels, water limitation (dry area: Supplementary Data), and nutrient-poor soils (Table 1). These conditions are known to increase the production of phenolic compounds, which are protective metabolites that help cells deal with oxidative stress [11,26]. High-light intensity encourages the accumulation of phenolics as a defence against UV radiation [27], while drought stress enhances their production by increasing oxidative pressure [28].

Chlorogenic acid is a prominent hydroxycinnamic acid derivative that is composed of caffeic and quinic acid. It is widely distributed in medicinal and edible plants [29]. Its identification in *A. betulina* across all three geographical regions indicates that it is a constitutive metabolite in this species. A previous study reported the presence of chlorogenic acid in *A. crenulata* but not in *A. betulina* [30]. Its consistent presence across all sampling sites suggests that chlorogenic acid may play a fundamental physiological or ecological role in the species, potentially related to defence mechanisms against oxidative stress or herbivory.

Pharmacologically, chlorogenic acid is among the most studied phenolic acids due to its wide-ranging bioactivities. It is known to exert potent antioxidant activity by scavenging free radicals and modulating oxidative pathways [31]. In addition, it has demonstrated antiviral effects (including against influenza and hepatitis), antidiabetic properties via an inhibition of glucose absorption and a modulation of glucose metabolism, and anti-inflammatory effects through a suppression of pro-inflammatory cytokines [32]. It also exhibits protective cardiovascular effects by improving lipid profiles and endothelial functions [33]. The presence of chlorogenic acid in *A. betulina* supports the ethnomedicinal use of this plant and underscores its potential in the development of health-promoting formulations.

In this study, 4-hydroxybenzoic acid 4-(6-O-sulfo) glucopyranoside reached its highest concentration in Citrusdal samples. This may suggest either an adaptive chemical response to the microclimate or an inherent metabolic tendency of *A. betulina* in that region. Notably, sulfated phenolics are rarely reported in the *Agathosma* species, making this finding both novel and potentially significant for future chemotaxonomic or pharmacological investigations.

#### 2.2.2. Flavonoids

Flavanols and various flavonoid derivatives were among the key subclasses of the flavonoids that were identified in the current study, as presented in Table 2 and Figure 2. Flavonoids represented a major component of the phenolic profile of *A. betulina*, and included a variety of flavonols (ten) such as rutin, quercetin 3-galactoside, disomin, hesperidin, quercetin 3-glucosyl-(1->2)-galactoside, trifolin, eupatolitin 3-galactoside, nicotiflorin, astragalin 7-rhamnoside, and isorhamnetin, as well as three other flavonoid-related compounds including apiin, cypellogin A, and icariside F2.

Semi-quantitative analysis revealed distinct regional variations in flavonoid distribution across the three geographic areas. Quercetin 3-galactoside (162.31 mg/g), quercetin 3-glucosyl-(1->2)-galactoside (10.27 mg/g), astragalin 7-rhamnoside (130.98 mg/g), trifolin (45.41 mg/g), cypellogin A (189.58 mg/g) and apiin (46.33 mg/g) were the most abundant in the samples from the Citrusdal Mountains, highlighting the prominence in flavonoid biosynthesis in the plants from this region. In contrast, isorhamnetin (24.16 mg/g), eupatolitin 3-galactoside (8.68 mg/g), diosmin (30.92 mg/g), rutin (1036.92 mg/g), and icariside F2 (17.28 mg/g) were the highest in the Groot Winterhoek region. The highest concentration of nicotiflorin (279.12 mg/g) and hesperidin (589.60 mg/g) was recorded in samples from the Cederberg mountains.

The most prominent finding was the exceptionally high concentration of hesperidin in the samples from the Cederberg region, which represented the highest concentration among the flavonoids identified. Hesperidin is a citrus-derived flavanone glycoside. It is widely recognised for its antioxidant, anti-inflammatory, and vasoprotective properties [34]. Diosmin and hesperidin are also widely recognised for their therapeutic effects, such as treating chronic venous insufficiency and haemorrhoids [35,36]. Rutin was another important compound that was identified. It strengthens blood vessel walls and reduces capillary fragility, making it useful in treating diseases that are associated with oxidative stress and inflammation [8,37]. The presence of these flavonoids in *A. betulina* supports the plants’ traditional use in treating conditions related to vascular and inflammatory disorders.

Quercetin 3-galactoside, identified in high amounts in *A. betulina* from Citrusdal, is a glycosylated flavonoid known for its strong antioxidant, anti-inflammatory, and anti-diabetic properties [38,39]. Quercetin 3-galactoside has previously been reported in the vegetative parts of *Azadirachta indica*, a medicinal plant from the family *Meliaceae* [40]. The glycosylation of quercetin, as seen in quercetin 3-galactoside, enhances its chemical stability, solubility, and bioavailability, thereby improving its therapeutic potential, particularly in the management of diabetes and cancer [41,42]. Its presence in this study highlights the therapeutic potential of *A. betulina*, particularly for managing oxidative stress and metabolic disorders.

The elevated flavonoid concentrations observed in Citrusdal samples can be attributed to improved soil fertility, particularly higher nitrogen, phosphorus, and soil organic matter content. These conditions enhance primary metabolic processes such as photosynthesis and carbon assimilation, thereby increasing the availability of the precursor molecules that are required for the phenylpropanoid pathway. Consequently, plants growing under nutrient-enriched conditions can allocate more resources toward the biosynthesis of flavonoids, which function in UV protection, antioxidative defence, and signalling [43].

#### 2.2.3. Terpenoid

##### Iridoids Glycoside

Nine iridoids and their derivatives were detected in the current study, as illustrated in Table 2 and Figure 2. These included: aucubin, hydroxyornoside, secologanin, epi-12-palmatoside G, sinocrassoside B3, fischeroside C, veranisatin C, lucidumoside C, and anacheiloside compounds that are known to play significant roles in plant defence and human health. The semi-quantitative analysis revealed that secologanin (81.50 mg/g), epi-12-palmatoside G (142.26 mg/g), fischeroside C (60.63 mg/g), sinocrassoside B3 (71.54 mg/g), and hydroxyornoside (40.13 mg/g) were highly concentrated in the samples collected from the Groot Winterhoek region. Conversely, anacheiloside (13.76 mg/g), veranisatin C (109.81 mg/g), lucidumoside C (5.01 mg/g), and aucubin (6.64 mg/g) were predominantly accumulated in the Citrusdal samples.

Iridoids are a class of monoterpenoids with wide-ranging biological activities, including anti-inflammatory, antioxidant, hepatoprotective, cardioprotective, and neuroprotective effects [44]. Secologanin is a key precursor in the biosynthetic pathway of various alkaloids, and its high concentration in Groot Winterhoek emphasises the chemotaxonomic and medicinal value of *A. betulina* from this site. Similarly, aucubin and hydroxyornoside have been studied for their roles in liver protection and their inhibition of reactive oxygen species [45]. The compound, epi-12-palmatoside G, was reported in *A. betulina* across all three sampling sites, signalling a landmark first-time report of this compound in this species. Epi-12-palmatoside G has previously been isolated from *Fibraurea tinctoria*, a medicinal plant used in traditional Southeast Asian medicine [46]. Pharmacological studies have shown that this compound exhibits inhibitory effects on nitric oxide (NO) production in lipopolysaccharide-stimulated macrophages, indicating promising anti-inflammatory activity [46]. This mode of action suggests that epi-12-palmatoside G may modulate immune responses and could be of pharmacological interest for treating inflammatory conditions [46]. The discovery of epi-12-palmatoside G in *A. betulina* expands the known phytochemical repertoire of this medicinal species and highlights its potential for yielding novel bioactive agents. Its relatively high concentrations, especially in Groot Winterhoek, further suggest that this region may offer optimal conditions for the biosynthesis of iridoid or secoiridoid-like glycosides.

Based on information provided by the Cape Nature ranger at the Groot Winterhoek site, the area from which the samples were collected had experienced a fire in the previous year. This recent fire incident may have contributed to the elevated levels of bioactive compounds that were observed in samples from this location. Fire is a fundamental ecological driver in fynbos ecosystems, playing a critical role in nutrient cycling, species regeneration, and secondary metabolite production [47]. The stimulation of phenolic and other bioactive compound synthesis following fire exposure is well documented in fire-adapted plant communities like fynbos, as plants often respond to post-fire stress by enhancing their chemical defences, and the compounds identified in this area are mainly useful in plant defence [48]. Furthermore, the presence of veranisatin C, a compound known for its neurotoxic and insecticidal properties, was found in high concentrations in Citrusdal. This could indicate a unique ecological interaction in this region, perhaps involving insect predation or specific microbial communities.

##### Terpenoid Derivative

As presented in Table 2 and Figure 2, a diverse range of secondary metabolites was identified, including terpenoid derivatives. Notably, samples from the Groot Winterhoek region exhibited the highest concentrations of loliolide β-D-glucopyranoside (6.31 mg/g) and tetraludin A (83.17 mg/g). The elevated accumulation of these compounds may be attributed to the environmental conditions that are characteristic of this region, including high irradiance, periodic water limitation, and nutrient-poor soils, which are known to stimulate terpenoid biosynthesis as part of plant adaptive responses [11]. From a biochemical perspective, terpenoids such as tetraludin A are widely recognised for their diverse pharmacological properties, including antimicrobial, anti-inflammatory, anticancer, and immunomodulatory activities, which contribute to plant defence and ecological fitness [49]. Similarly, loliolide derivatives have been reported to exhibit antioxidant and cytoprotective effects, functioning as stress-responsive metabolites that mitigate oxidative damage under adverse environmental conditions [26]. Therefore, the higher concentrations of these terpenoid derivatives in the Groot Winterhoek samples likely reflect an adaptive metabolic response to environmental stress, enhancing both plant survival and the medicinal value of the species.

#### 2.2.4. Saponins

As illustrated in Table 2 and Figure 2, the saponin identified in this study was neosaponarin, which exhibited significant variations in concentration across the different geographic locations, underscoring site-specific influences on its biosynthesis and accumulation. Neosaponarin was found to be the most abundant in samples collected from Citrusdal (10.03 mg/g), suggesting favourable environmental conditions in this region for its synthesis.

#### 2.2.5. Vitamins/Cofactors

Riboflavin (vitamin B2) was the only vitamin identified in the current phytochemical analysis of *A. betulina* (Table 2, Figure 2). Semi-quantitative data revealed a clear variation in its distribution across the three collection sites, with the highest concentration detected in samples from Citrusdal (1.93 mg/g), followed by Cederberg (1.24 mg/g), and the lowest concentration detected in Groot Winterhoek. Riboflavin is an essential water-soluble vitamin that is involved in various cellular processes, including energy production, redox reactions, and the metabolism of fats, drugs, and steroids [50]. Its presence in *A. betulina* adds to the plants’ nutritional and therapeutic profile. The significantly higher riboflavin levels in Citrusdal suggest that this location provides more favourable environmental or ecological conditions, such as optimal soil nutrients or light exposure for vitamin biosynthesis or retention. These findings further support Citrusdal as a highly suitable region for the cultivation of *A. betulina* because of its enhanced nutritional soil quality.

#### 2.2.6. Steroids/Steroidal Derivatives

The current study identified 5α-androstan-3α, 17β-diol disulfate as the sole steroidal compound present in *A. betulina* across the sampled sites. The analysis showed a distinct geographical variation in its accumulation. The highest concentration was found in Citrusdal (3.88 mg/g), followed by Groot Winterhoek (1.66 mg/g), and the lowest concentration was found in Cederberg (0.73 mg/g). This compound, a sulfated metabolite of dihydrotestosterone (DHT), is known for its role in androgen metabolism and its potential regulatory effects in steroid hormone pathways [51].

#### 2.2.7. Oligosaccharides/Polysaccharide Derivatives

Across the three sampling sites, the current study revealed the presence of two significant oligosaccharides and/or polysaccharide derivatives (Table 2): Cyclobis-(1>6)-α-nigerosyl and CHEBI:68582 (β-D-Galp-(1>3)-[α-L-Fucp-(1>4)]-β-D-GlcpNAc-(1>3)-β-D-Galp-(1>3)-[α-L-Fucp-(1>4)]-β-D-GlcpNAc). Cyclobis-(1>6)-α-nigerosyl, a cyclic oligosaccharide composed of α-1,6-linked glucose units, showed its highest accumulation in Citrusdal (5.23 mg/g), with lower levels in Groot Winterhoek (2.80 mg/g) and minimal presence in Cederberg (0.73 mg/g). This distribution may reflect site-specific enzymatic activity related to starch breakdown or microbial interactions that influence carbohydrate metabolism. In contrast, the complex fucosylated polysaccharide derivative, structurally related to the glycan motifs that are found in glycoproteins and known for their potential immunomodulatory effects [52], was found in the highest concentration in Groot Winterhoek (88.53 mg/g). The pronounced abundance of this compound in that region suggests that environmental factors such as temperature, soil nutrients, fire, or microbial symbiosis may be promoting its biosynthesis or accumulation.

### 2.3. Multivariate Analysis

**Figure 3:**
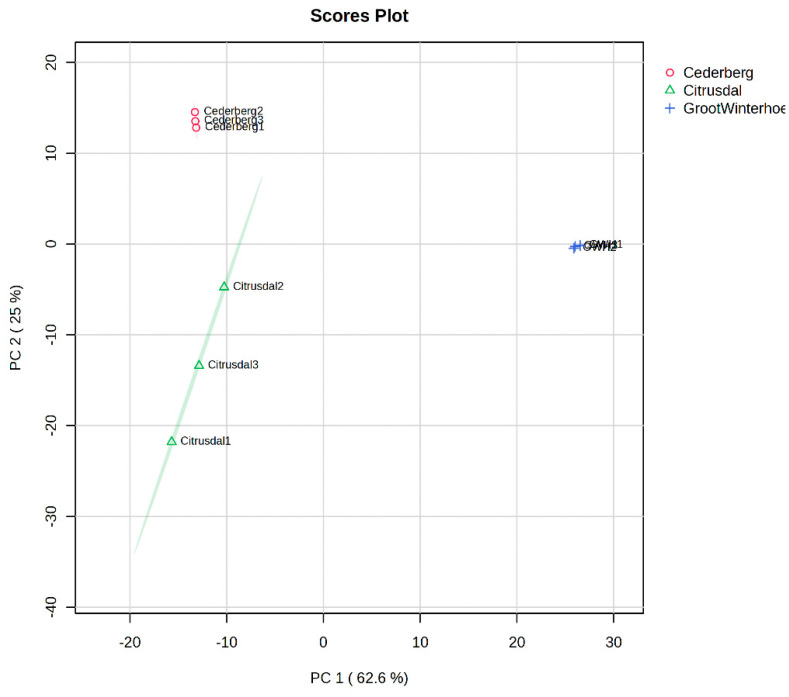
Scatter plot scores based on principal component analysis. PC1 vs. PC2 scores of *A. betulina* showing separation within the three locations.

Samples were analysed with an unsupervised principal component analysis (PCA) based on profiling (obtained from the UPLC-QTOF-MS), which indicated a preliminary understanding of the overall metabolites that were distributed differently and the magnitude of the variability between the samples within the groups. According to the PCA plot, three separate clusters were determined by the types of secondary metabolites that were exhibited in *A. betulina* leaves from different geographic locations (Figure 3). This aligns with previous findings that environmental and edaphic factors can influence the metabolite profiles in medicinal plants [53]. The small sample size in this study was insufficient for the development of robust chemometric models. Because the goal was not to develop a prediction model but simply to explore intra-species variation, chemometric analysis was nonetheless applied to the LC-MS data. These two PC models were able to give a clear separation of the locations: 25% separation along PC2 and 62.6% separation along PC1, respectively, thereby explaining 87.6% of the total variability of the dataset. The spatial distribution of the samples in the PCA score plot revealed clear grouping according to the region, suggesting distinct compositional or trait-based differences. Samples from Cederberg were tightly clustered in the upper-left quadrant (positive PC2, negative PC1), indicating homogeneity within the group and a shared response to the variables contributing to this principal component space. In contrast, Citrusdal samples were more dispersed, primarily located along the negative axis of both PC1 and PC2, which reflects greater intra-regional variability. This variability may be indicative of environmental heterogeneity or phenotypic plasticity within the Citrusdal region.

The Groot Winterhoek samples are clearly separated from the other two groups, occupying a position far along the positive side of PC1 and near-zero on PC2. This marked separation suggests that the variables loading strongly on PC1 are highly influential in defining the characteristics of Groot Winterhoek samples, setting them apart from those of Cederberg and Citrusdal. The loading vectors (green arrow/line) represent the contribution of individual variables to the PCs. Their direction and magnitude indicate the traits or chemical constituents most responsible for the observed differentiation, with longer arrows pointing toward the most discriminative variables.

**Figure 4:**
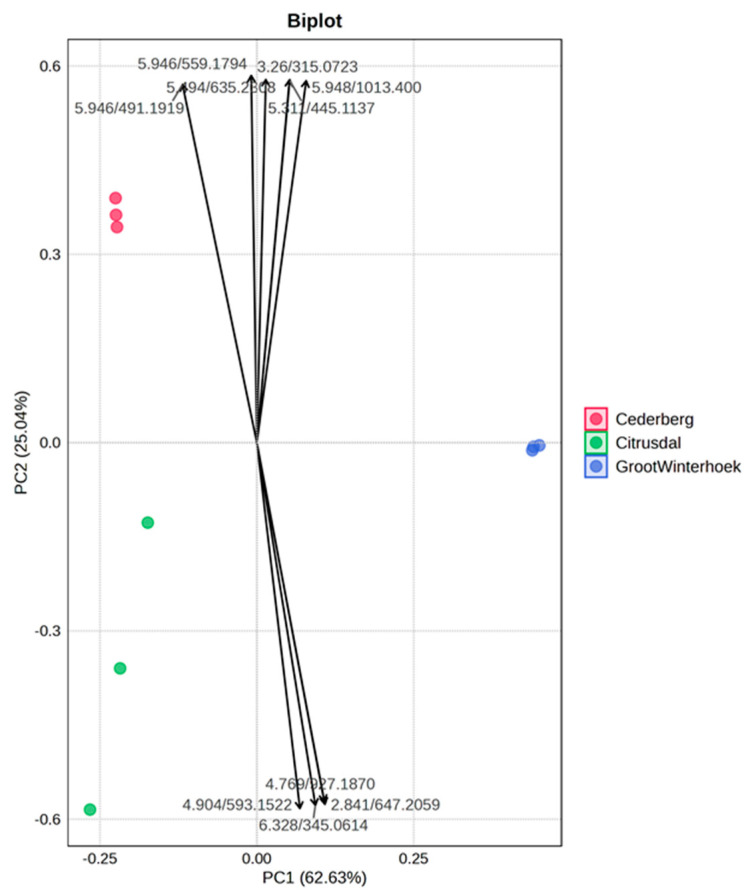
The principal component analysis (PCA)—biplot of identified secondary metabolites based on the variance across locations.

The PCA biplot (Figure 4) reveals clear regional differentiation among samples from Cederberg, Citrusdal, and Groot Winterhoek, with PC1 (62.63%) and PC2 (25.04%) together explaining a significant 87.67% of the total variance. Cederberg samples cluster tightly along the positive PC2 axis, suggesting a strong internal similarity and association with specific variables that load heavily in that direction. Citrusdal samples are more dispersed along the negative sides of both components, indicating a higher variability and a weaker correlation with the primary contributing variables. In contrast, the Groot Winterhoek samples are distinctly separated along the positive PC1 axis, influenced by variables such as 5.948/1013.400 and 5.946/559.1794, which strongly contribute to their unique profile. The loading vectors confirm that different sets of traits or chemical constituents drive the separation among regions, emphasising the significant effect of geographic origin on the phenotypic or phytochemical characteristics of the sample.

**Figure 5:**
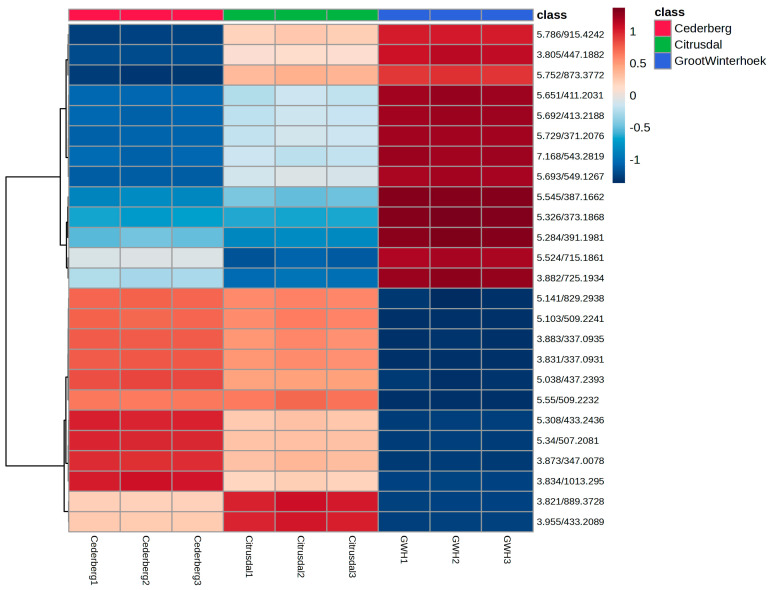
Heatmap of *A. betulina* secondary metabolites that were analysed via UPLC-QTOF-MS.

The heat map (Figure 5) was tentatively derived from untargeted compounds. These provide a visual representation of the clustering and relative abundance or expression of the variables across the three regions, and Cederberg, Citrusdal, and Groot Winterhoek and support the patterns observed in the PCA and biplot analyses. The Cederberg samples (red) form a distinct cluster with consistently high values (indicated by warm colours) for a specific subset of variables, confirming their tight clustering in the PCA and alignment with certain principal components in the biplot. In contrast, the Citrusdal samples (green) display greater heterogeneity with moderate expression levels across a broader range of variables, which is consistent with their scattered positioning in the PCA space and their weaker loadings in the biplot [54].

The Groot Winterhoek samples (blue) exhibit a unique cluster characterised by distinct variable patterns, mostly dominated by a low expression (cool colours) for variables that are highly expressed in other regions. This further explains their strong separation along PC1 in the PCA and their alignment with dominant loading vectors in the biplot. Overall, the heat map reinforces the multivariate distinction among the three regions, highlighting region-specific biochemical or trait signatures that drive the observed clustering and principal component contributions. This implies that environmental conditions, genetic factors, or both may be driving the variation that has been observed. The distinct clustering of the Groot Winterhoek samples suggests the presence of unique traits or compounds that require further investigation. The elevated metabolite levels that are observed in Groot Winterhoek may be attributed to a recent wildfire incident in the area, which is known to induce stress-related phytochemical responses in plants.

Although clear regional clustering was observed, the present study did not quantify site-specific environmental parameters such as soil pH, mineral nutrient composition, or microclimatic humidity. Therefore, causal relationships between specific abiotic factors and metabolite accumulation cannot be established. Future research integrating environmental covariates with metabolomic profiling using multivariate regression or mixed-effects modelling would allow the partitioning of the variance to be attributable to specific ecological drivers.

## 3. Materials and Methods

### 3.1. Chemicals

Reagents and solvents that were utilised in this study included methanol (CH_3_OH), Romil LCMS grade; formic acid (HCOOH), ≥98%; and acetonitrile (CH_3_CN), Romil LCMS grade, which were obtained from Sigma-Aldrich, Johannesburg, South Africa.

### 3.2. Collection of Plant Material and Soil Sampling

**Figure 6:**
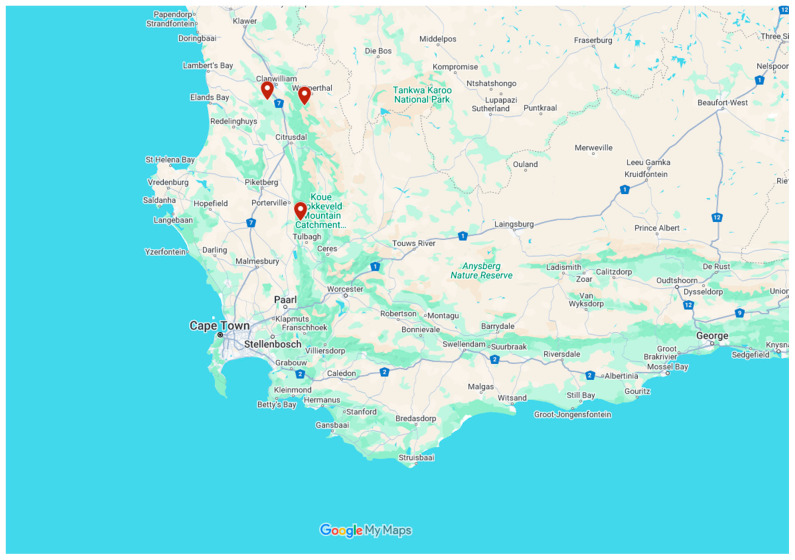
Map of South Africa showing the sampling sites in the Western Cape Province (Google Maps, 2025).

The wild *A. betulina* leaves used in this study were collected from three Western Cape mountainous sites located within Cape Nature conservation areas and private land in South Africa. These sites were visited in January 2025 and are as follows: Groot Winterhoek Wilderness (32°59′53.5″ S, 19°03′29.0″ E), Skimmelberg Mountain in Citrusdal (32°19′44.4″ S, 18°46′55.05″ E), and the Algeria section of the Cederberg mountains (32°21′28.3″ S, 19°02′17.0″ E) (Figure 6). A trained ranger from Cape Nature helped with the identification and collection of the samples. The samples from the Cederberg and Citrusdal were collected from steeper south-facing slopes, while those from Groot Winterhoek were obtained from a flatter, but still south-facing, area. All three study sites fall within a winter rainfall region. A total of 15 kg of plant material was collected, adhering to Cape Nature’s ethical harvesting protocols within a marked 3-hectare area. Ten plants were randomly selected within the plot. Samples were taken from each plant, and a single pooled sample was prepared from all of the plants. The harvested material was placed into sampling bags and promptly transported to the Agricultural Research Council (ARC) Infruitec-Nietvoorbij for processing.

Upon arrival, leaves were separated from the twigs and air-dried at room temperature for five days until a consistent weight was achieved. This method was used to retain the natural green colouration and prevent discolouration on the leaves. The dried samples were finely ground and stored in glass vials at 4 °C for subsequent analysis. Voucher specimens (B01, B02 and B03) were prepared and retained at the ARC Infruitec-Nietvoorbij, Stellenbosch, South Africa. The soil samples were collected from the study sites using the zig-zag sampling method, where ten subsamples were taken with a soil auger from a depth of 30 cm to form a representative sample (per site). The samples were then stored in plastic bags for subsequent drying, sieving, and testing. Soil nutrient analyses, including phosphorus, potassium, magnesium, manganese, zinc, calcium, and sodium, were conducted at Bemlab, located in Somerset West, Cape Town. The available phosphorus was determined using the Bray and Kurtz (1945) [55] method, while cation exchange capacity (CEC) and exchangeable bases were measured using the ammonium acetate extraction method. The soil pH was determined in a soil–water suspension (1:10, *w*/*v*) using a calibrated pH metre.

### 3.3. Methanolic Extraction of Samples

Approximately 0.25 g of the dried and finely ground leaves of the *A. betulina* plant material was accurately weighed into a 15 mL Falcon tube and extracted with 10 mL 50% methanol/1% formic acid in water. This was done on 9 samples (each location sample replicated 3 times). The extraction was carried out using an E-UC13-HD-D Eins Sci ultrasonic bath (Sigma-Aldrich, Johannesburg, South Africa) set at 300 W and 35 °C for 30 min. After sonication, the mixture was continuously agitated on a shaker for 24 h. To separate plant debris, the extract was vortexed and then centrifuged at 5000× *g* for 10 min using a Hermle Z160M centrifuge (Hermle LaborTechnik, Wehinger, Germany). The resulting supernatant was filtered through a 0.22 μm Millipore membrane filter (Merck^®^ Millipore, Johannesburg, South Africa) and stored in a glass vial at −20 °C for subsequent UPLC-QTOF-MS analysis.

### 3.4. UPLC-QTOF-MS Analysis Method

A Waters Cyclic Quadrupole time-of-flight (qTOF) mass spectrometer (MS) connected to a Waters Acquity ultra-performance liquid chromatograph (UPLC) (Waters, Milford, MA, USA) was used for high-resolution UPLC-MS analysis using the methods reported by Lai et al. (2018) and Tsugawa et al. (2015) [56,57]. The column eluate was first detected by a photodiode array (PDA) prior to going through the MS, thus allowing for the simultaneous collection of MS and UV spectra. In negative mode, electrospray ionisation was used with a desolvation gas at 800 L/h and 275 °C and a cone voltage of 20 V. The rest of the MS settings were optimised for the best sensitivity and resolution. In MSE mode, data were obtained by scanning from 100 to 1500 *m*/*z*. Two channels of MS data were obtained in MSE mode, i.e., at 4 V (low collision energy) and 40–100 V (collision energy ramp) to acquire fragmentation data. For accurate mass determination, leucine enkaphalin was used as a reference mass/lock mass while sodium formate was used to calibrate the instrument. To achieve separation, the Waters HSS T3 instrument was set at 2.1 × 150 mm, 1.7 μm column. The mobile phase comprises 0.1% formic acid as solvent (B), as well as 0.1% containing acetonitrile as solvent (A) in a combined volume of 2 μL. In a linear way, the gradient commenced at 100% for 0.5 min with solvent A and shifted to 44% for solvent B over 7 min.

Consequently, the gradient progressed in solvent B to 40% over 50, then a wash step of 12 min at 100% B, then re-equilibration to the initial conditions for 1 min. The column temperature was maintained at a constant 60 °C, and the flow rate was set at 0.35 mL/min throughout the analysis. Quantification of the metabolites was carried out in a semi-quantitative manner using a calibration curve that was constructed from catechin standards ranging from 0.5 to 100 mg/L, allowing for the relative estimation of compound concentrations. The acquired UPLC-QTOF-MS data were processed using MS-DIAL (version 5.0) and MS-FINDER (version 3.5) software tools, developed by the RIKEN Centre for Sustainable Resource Science (Metabolome Informatics Research Team, Kanagawa, Japan) [57,58]. These platforms enabled comprehensive metabolite deconvolution, peak alignment, annotation, and structure prediction, facilitating the accurate identification of both known and novel compounds in the sample matrix.

### 3.5. Chemometric Data Analysis

The UPLC-QTOF-MS data were subjected to multivariate analysis using principal component analysis (PCA) to identify key discriminatory variables. Pre-processing of the raw data, including alignment, peak detection, and filtration, was performed using MarkerLynx v4.1. The analytical parameters were set to a mass range of 100–1000 Da and a retention time window of 5–21 min, with a mass tolerance of 50 mDa. In addition, a 0.4 min retention time tolerance, a 500-intensity threshold/counts of collection parameters, and a noise elimination level of 1.00 were all set. The resulting peak data, comprising *m*/*z* and retention time pairs, were imported into SIMCA-P+ (version 13.0; Umetrics, Umeå, Sweden) for chemometric analysis and the construction of multivariate statistical models to explore relationships within the dataset.

## 4. Conclusions

This study profiled and quantified secondary metabolites from three distinct mountainous regions in the Western Cape Province of South Africa to understand the distribution of compounds in relation to their geographical locations. The observed geographic variation in metabolite profiles reflects the underlying metabolic trade-offs between growth and defence, likely driven by site-specific environmental conditions. In resource-rich environments such as Citrusdal, plants can allocate sufficient resources to both their growth and to the biosynthesis of energetically demanding compounds such as flavonoids, which are supported by higher nutrient availability and enhanced metabolic activity. In contrast, resource-limited and stress-prone environments such as Groot Winterhoek promote a shift in metabolic allocation toward defence-related compounds, particularly iridoid glycosides, which enhance plant resilience under conditions of environmental stress, including nutrient limitation, drought, and post-fire disturbance. The Cederberg mountains favoured the accumulation of phenolic acids, while the enhanced nutrient availability and moderate stress conditions in Citrusdal likely promoted carbon allocation toward flavonoid biosynthesis via the phenylpropanoid pathway in the Citrusdal region.

Consequently, geographic location plays a critical role in shaping the secondary metabolite composition of *Agathosma betulina*, with variations in soil fertility, acidity, and microclimatic conditions collectively influencing phytochemical expression in *A. betulina*, which have direct implications on quality, yield, and conservation strategies. All three study sites are within a winter rainfall region, indicating that precipitation patterns alone are unlikely to be the main factor influencing the variation in phenolic composition.

Overall, the findings emphasise the ecological plasticity and chemotype variability of *A. betulina*, underscoring the importance of environmental and spatial factors in shaping its phytochemical landscape. Given the comprehensive phytochemical richness and the elevated concentrations of pharmacologically relevant compounds, Citrusdal and Cederberg are recommended as the most favourable locations for the selection of chemotypes for geomatics and ultimately foster a sustainable cultivation of *A. betulina*. Citrusdal is also recommended for cultivation, as its environmental conditions appear optimal for supporting the biosynthesis and accumulation of a wide range of medicinally valuable metabolites, making it a strategic site for both conservation and commercial production.

The conservation of this important native ethnomedicinal plant is critical, and techniques such as micropropagation offer valuable tools not only for safeguarding the species but also for facilitating the sustainable biosynthesis of its bioactive compounds. Comprehensive, long-term investigations are required to gain a deeper understanding of the polyphenolic compound accumulation in this species. Mapping the geographical distribution of these secondary metabolites could yield valuable insights with implications for therapeutic, industrial, pharmaceutical, and agricultural applications, including the development of novel organic drugs, agrochemicals, and functional food products.

## Figures and Tables

**Table 1:** Physical and chemical properties of topsoil (0–30 cm) of *A. betulina* harvest sites.

Parameters	Locations		
	Groot Winterhoek	Citrusdal (Skimmelberg)	Cederberg (Algeria)
**Physical properties**			
Type	Sand	Sand	Sand
Water holding 10 kPa (%)	29.20	***	21.6
Water holding 100 kPa (%)	16.45	***	12.05
Water holding mm/m (%)	127.53	***	90.15
**Chemical properties**			
pH KCl	3.7	4.4	4.4
H^+^ cmol ((+)/kg)	1.13	1.26	1.04
NO_3_^−^ (mg/kg)	<0.36	2.3	0.82
P (Bray ll) (mg/kg)	6.3	15.1	6.3
NH_4_^+^ (mg/kg)	2.3	8.5	4.6
C (WB) (%)	1.02	1.69	0.72
SOM (%)	1.76	2.91	1.23
S Am.acet (mg/kg)	<3.4	5.3	<3.4
CEC (cmol/kg)	1.37	2.99	3.50
(Na^+^) ^a^ (%)	2.66	1.86	6.46
(K^+^) ^a^ (%)	1.79	2.84	4.34
(Ca^+^) ^a^ (%)	23.97	47.83	33.33
(Mg^2+^) ^a^ (%)	11.72	13.82	20.41
T Value ^a^ (cmol/kg)	1.88	3.76	2.94
Acid Sat. ^a^ (%)	60.05	33.57	35.38

*** = Insufficient sample, ^a^ = Base Saturation, CEC = Cation Exchange Capacity, and SOM = Soil Organic Matter.

**Table 2:** Proximate identification of *Agathosma betulina* major compounds from different locations in the Western Cape using UPLC-QTOF-MS.

RT (Min)	[M-H]^−^ *m*/*z*	Tentative Identification	Class	MS/MS Fragment Ions (*m*/*z*)	References	Locations		
						Groot Winterhoek (mg/g)	Citrusdal (Skimmelberg) (mg/g)	Cederberg (Algeria) (mg/g)
2.817	451.14737	5α-Androstan-3α,17β-diol disulfate	Sulfated steroids	292.8118, 295.10446, 371.77264, 451.14618	PubChem	1.66	3.88	0.73
2.841	647.20599	cyclobis-(1->6)-α-nigerosyl	Oligosaccharides	238.8918, 275.85367, 327.10883, 329.08826, 340.7926	PubChem	2.80	5.23	0.73
2.972	375.13071	Riboflavin	Flavins	292.81244, 337.0939, 371.77072	PubChem	0.36	1.93	1.24
3.017	379.03583	4-hydroxybenzoic acid 4-(6-O-sulfo)glucopyranoside	Phenolic glycosides	265.07745, 292.81161, 295.10376, 371.77048, 379.03455	[15]	1.60	4.32	1.52
3.26	315.07233	gentesic acid 5-O-glucoside	Phenolic glycosides	292.81137, 309.81393, 315.07175	PubChem	25.48	16.52	29.03
3.362	345.11996	Aucubin	Iridoid O-glycosides	191.05569, 292.81198, 309.81482	PubChem	3.09	6.64	2.66
3.586	353.0878	Chlorogenic acid	Quinic acids and derivatives	191.05629, 242.05797, 243.0661, 271.0983	[16]	7.39	5.82	14.65
3.723	401.14661	Icariside F2	O-glycosyl compounds	191.05696, 271.10019, 292.81125, 309.8143, 371.09998	PubChem	17.28	5.49	7.52
3.77	387.12991	Secologanin	Terpene glycosides	179.07124	[17]	81.50	34.91	33.09
4.046	593.15302	Neosaponarin	Flavonoid-7-O-glycosides	238.89235, 313.13031, 340.7915, 347.17172, 349.18646	PubChem	3.04	10.03	3.80
4.106	625.14246	Quercetin 3-glucosyl-(1->2)-galactoside	Flavonoid-3-O-glycosides	238.89235, 313.13031, 340.7915, 347.17172, 349.18646	PubChem	3.66	10.27	1.57
4.187	583.20428	Lucidumoside C	Terpene glycosides	179.05571, 238.89276, 300.1095, 327.12344, 353.08945	PubChem	3.13	5.01	0.25
4.283	625.17694	Anacheiloside	Flavonoid-3-O-glycosides	211.09845, 267.09113, 317.0661, 340.79279, 345.15573	PubChem	2.67	13.76	1.86
4.311	563.14178	Apiin	Flavonoid-7-O-glycosides	183.10291, 191.05621 197.11871, 201.11244, 225.11375	PubChem	10.18	46.33	13.31
4.498	357.15573	Loliolide β-D-glucopyranoside	O-glycosyl compounds	121.02956, 173.0446, 249.08009, 269.06702, 295.11804	PubChem	6.31	0.96	0.39
4.53	371.09863	Veranisatin C	Terpene lactones	191.05571, 199.09752, 249.0804, 3250.08353, 267.09079	PubChem	18.99	109.81	35.90
4.615	609.14636	Rutin	Flavonoid-3-O-glycosides	301.03537	[16,18]	1036.92	752.82	418.20
4.772	463.08875	Quercetin 3-galactoside	Flavonoid-3-O-glycosides	301.03488	[19]	96.62	162.31	28.77
4.904	593.15222	Astragalin 7-rhamnoside	Flavonoid-7-O-glycosides	345.10904, 347.1369, 355.82019, 358.06931, 359.07605	PubChem	59.93	130.98	15.88
5.072	447.09348	Trifolin	Flavonoid-3-O-glycosides	284.03275	[16]	24.06	45.41	4.99
5.195	609.18262	Hesperidin	Flavonoid-7-O-glycosides	300.05823	[20]	480.80	584.14	589.60
5.21	607.16772	Diosmin	Flavonoid-7-O-glycosides	286.04755, 301.03214	[21]	30.92	12.21	8.05
5.346	465.17725	Tetraludin A	Germacranolides and derivatives	137.02429, 163.04094, 165.0556, 175.04005, 193.05103	PubChem	26.12	81.19	60.77
5.415	507.11472	Eupatolitin 3-galactoside	Flavonoid-3-O-glycosides	135.04485, 175.04001, 179.03465, 191.05617, 193.04977	PubChem	8.68	2.74	1.24
5.494	567.24469	fischeroside C	Diterpene glycosides	179.07147, 195.06549, 239.09225, 254.21902, 255.02879	[22]	60.63	44.50	55.38
5.529	693.20422	Sinocrassoside B3	Flavonoid-7-O-glycosides	134.03746, 160.01666, 175.0397, 176.04369, 183.10181	PubChem	71.54	16.21	26.32
5.6	491.19247	epi-12-Palmatoside G	Naphthopyranone glycosides	119.05029, 163.04016, 164.04449, 175.04048, 205.05087	PubChem	142.26	132.26	120.54
5.672	519.18738	CHEBI:68582	Acylaminosugars	137.02417, 173.04527, 174.04881, 177.01952, 179.03546	PubChem	88.53	0.33	0.69
5.809	629.18848	Cypellogin A	Flavonoid-3-O-glycosides	301.03342, 463.08838	[23]	62.89	189.58	121.07
5.964	593.18762	Nicotiflorin	Flavonoid-7-O-glycosides	151.00465, 285.07684	[24]	130.90	189.31	279.12
6.329	315.05106	Isorhamnetin	Flavonols	238.89198, 253.21675, 271.02487, 300.02725, 301.03107	PubChem	24.16	16.32	2.25
6.356	643.20276	Hydroxyornoside	Macrolides and analogues	481.214	[25]	40.13	2.12	0.78

Values represent individual LC–MS measurements (n = 3 per sample) obtained for semi-quantitative comparison.

## Data Availability

The original contributions presented in this study are included in the article and Supplementary Materials. Further inquiries can be directed to the corresponding author.

## Supplementary Materials

The following supporting information can be downloaded at https://www.mdpi.com/article/10.3390/ijms27104486/s1.
