# Supplementary material for: Geographic Influence on Secondary Metabolite Profiles in Leaves of the Endemic Agathosma betulina (P.J. Bergius) Pillans. in the Western Cape Province, South Africa
Source: Int J Mol Sci. 2026 May 16;27(10):4486. doi: 10.3390/ijms27104486 (PMC13206881; doi:10.3390/ijms27104486)
Supplement: Supplementary file 1 [file ijms-27-04486-s001.zip › ijms-4160587-supplementary.pdf]

Supplementary Data

Average weather of the three locations

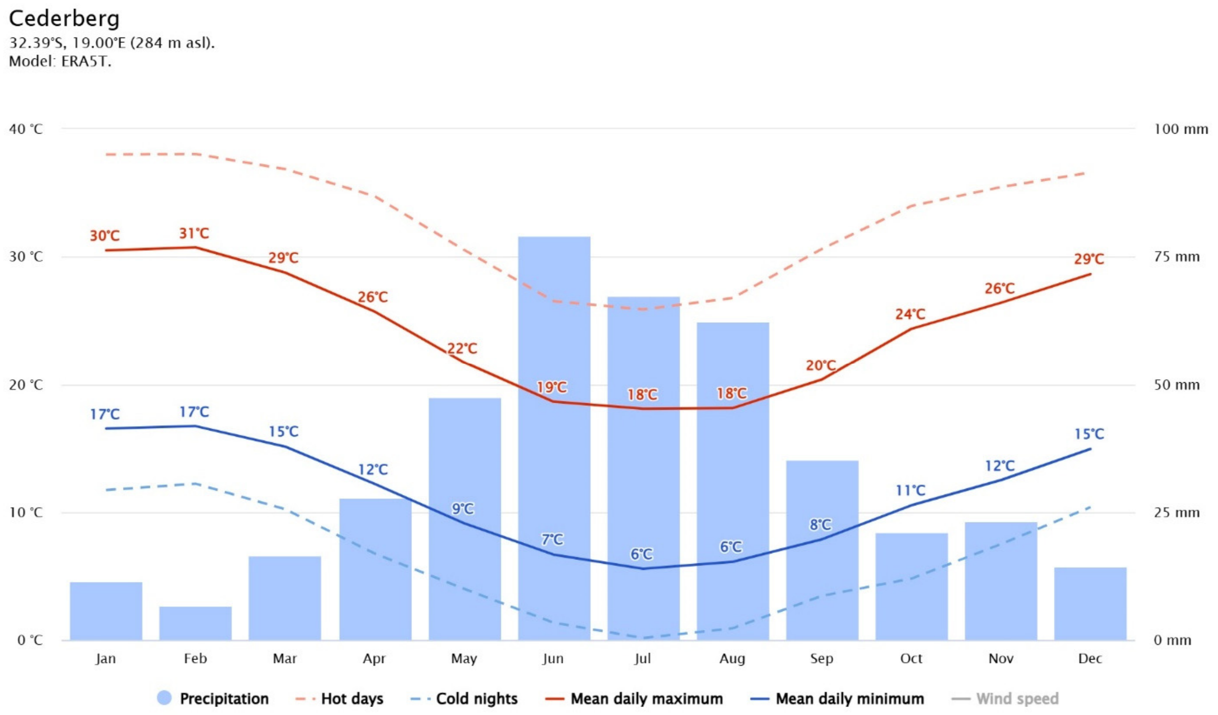

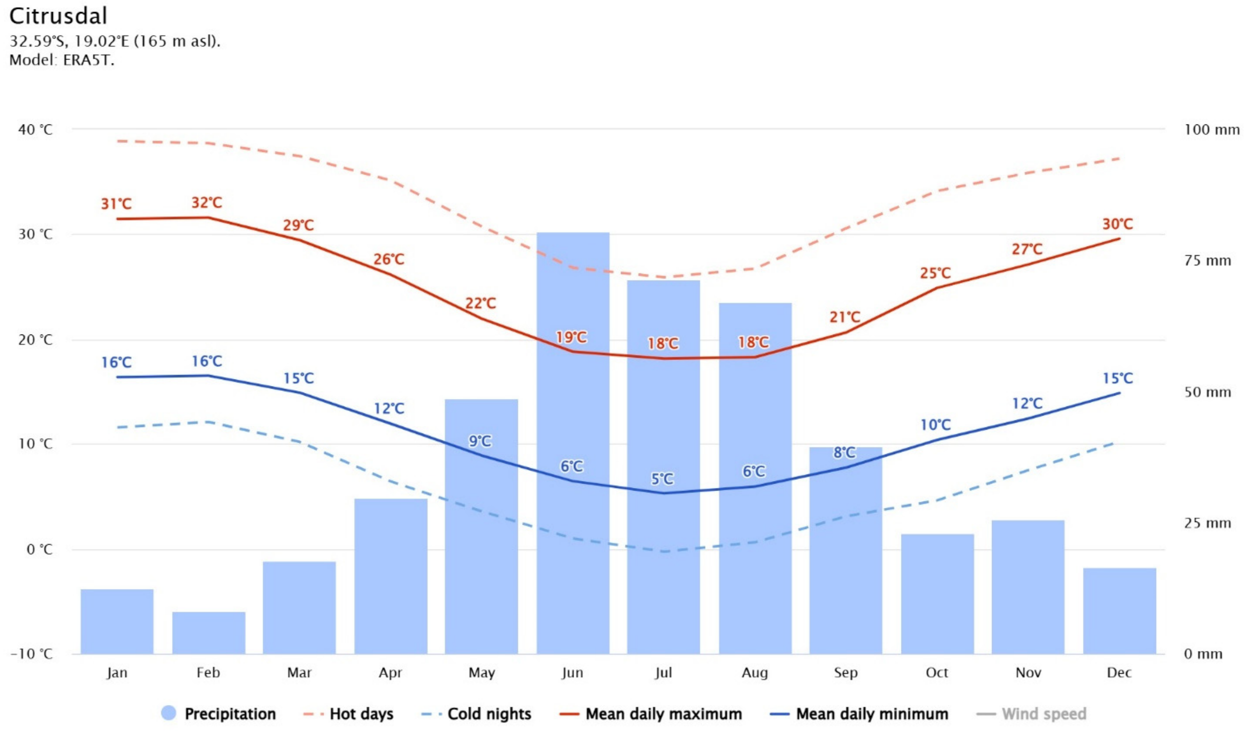

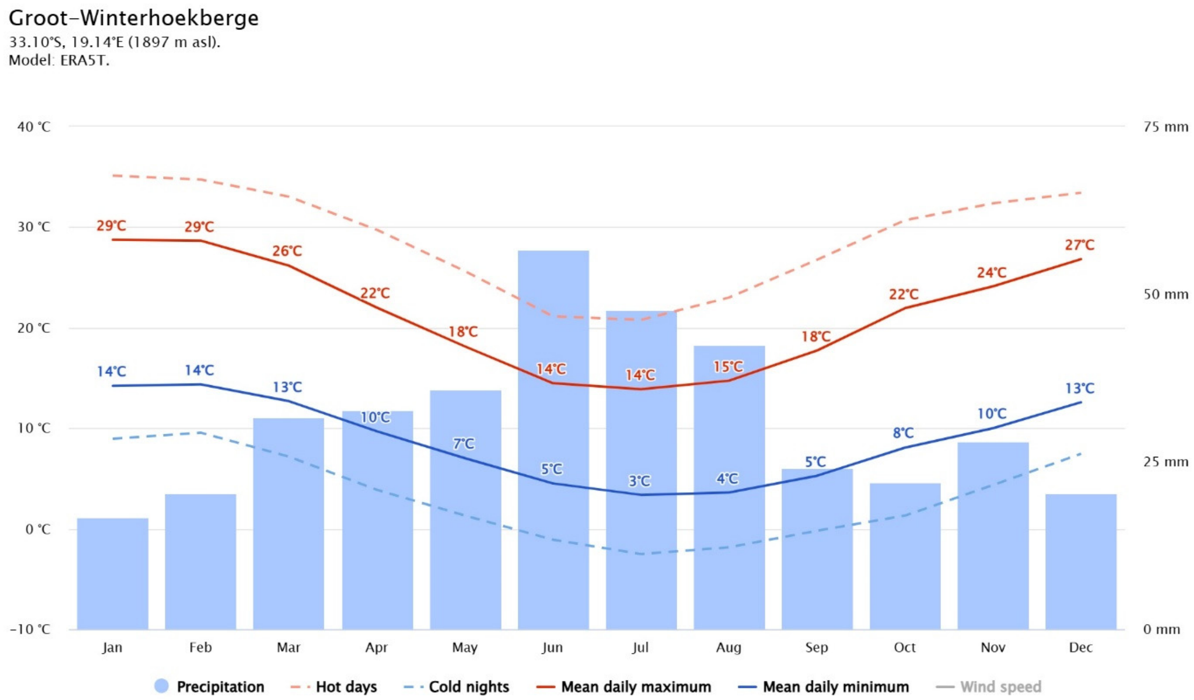

Source: metablue: https://www.meteoblue.com/en/weather/week/western cape_south-africa_3369103
